# Achieving Consensus for Management of Hormone-Sensitive, Low-Volume Metastatic Prostate Cancer in Italy

**DOI:** 10.3390/curroncol29070362

**Published:** 2022-06-28

**Authors:** Elena Verzoni, Giovanni Pappagallo, Filippo Alongi, Stefano Arcangeli, Giulio Francolini, Daniele Galanti, Luca Galli, Marco Maruzzo, Sabrina Rossetti, Giambattista Siepe, Luca Triggiani, Paolo Andrea Zucali, Rolando Maria D’Angelillo

**Affiliations:** 1 Department of Medical Oncology Unit, Fondazione IRCCS Istituto Nazionale dei Tumori, 20133 Milan, Italy; elena.verzoni@istitutotumori.mi.it; 2 School of Clinical Methodology, IRCCS “Sacro Cuore-Don Calabria” Hospital, Negrar di Valpolicella, 37024 Verona, Italy; giovanni.pappagallo@icloud.com; 3 Advanced Radiation Oncology Department, Cancer Care Center, IRCCS Sacro Cuore Don Calabria Hospital, 37024 Verona, Italy; filippo.alongi@unibs.it; 4 Department of Medical, Surgical and Health Care Specialties, University of Brescia, 25121 Brescia, Italy; luca.triggiani@unibs.it; 5 Department of Radiation Oncology, San Gerardo Hospital, 20900 Monza, Italy; stefano.arcangeli@unimib.it; 6 Radiation Oncology, Department of Medicine and Surgery University of Milan Bicocca, 20126 Milan, Italy; 7 Radiation Oncology Unit, Azienda Ospedaliero-Universitaria Careggi, 50134 Firenze, Italy; francolinigiulio@gmail.com; 8 Medical Oncology Unit, Buccheri La Ferla Fatebenefratelli Hospital, 90123 Palermo, Italy; galanti.daniele@fbfpa.it; 9 Division of Medical Oncology, Azienda Ospedaliero Universitaria Pisana, 56126 Pisa, Italy; l.galli@ao-pisa.toscana.it; 10 Oncology Unit 1, Istituto Oncologico Veneto IOV−IRCCS, 3512 Padova, Italy; marco.maruzzo@iov.veneto.it; 11 Department of Urology & Gynecology, Istituto Nazionale Tumori IRCCS Fondazione G Pascale, 80131 Naples, Italy; s.rossetti@istitutotumori.na.it; 12 Radiation Oncology, IRCCS Azienda Ospedaliero-Universitaria di Bologna, 40138 Bologna, Italy; giambattista.siepe@aosp.bo.it; 13 Department of Radiation Oncology, University and Spedali Civili Hospital, 25123 Brescia, Italy; 14 Department of Biomedical Sciences, Humanitas University, 20089 Milan, Italy; paolo.zucali@hunimed.eu; 15 Department of Oncology, IRCCS Humanitas Research Hospital, 20089 Milan, Italy; 16 Radiation Oncology, Department of Biomedicine and Prevention University of Rome “Tor Vergata”, 00133 Rome, Italy

**Keywords:** prostate, hormone-sensitive, low-volume, oligometastatic, ARTA, chemotherapy

## Abstract

Metastatic hormone-sensitive prostate cancer (mHSPC) is usually categorized as high- or low-volume disease. This is relevant because low- and high-volume metastatic disease are associated with different outcomes, and thus management of the two forms should differ. Although some definitions have been reported, the concept of oligometastatic disease is not so clearly defined, giving rise to further variability in the choice of treatment, mainly between systemic agents and radiotherapy, especially in the era of metastasis-directed therapy. With the aim of providing clinicians with guidance on best practice, a group of medical and radiation oncologists, experts in prostate cancer, used the round robin method to generate a series of consensus statements on management of low-volume mHSPC. Consensus was obtained on three major areas of controversy: (1) with regard to clinical definitions of mHSPC, it was held that oligometastatic and low-volume disease refer to different concepts and should not be used interchangeably; (2) regarding therapy of de novo low-volume metastatic disease, androgen deprivation therapy alone can be considered undertreatment, and all patients should be evaluated for systemic treatment combinations; local therapy should not be denied in patients with mHSPC, regardless of the intensity of systemic therapy, and metastasis-directed therapy can be proposed in selected cases; (3) with regard to treatment of metachronous metastatic disease, patients should be evaluated for systemic treatment combinations. Metastasis-directed therapy can be proposed to delay systemic treatment in selected cases, especially if prostate-specific membrane antigen positron emission tomography staging has been performed and when indolent disease occurs. It is hoped that clinicians treating patients with mHSPC in daily practice will find this expert opinion of value.

## 1. Introduction

Prostate cancer is the second most common cancer worldwide in men with over 1.4 million new cases in 2020 [1]. Recent data indicate that the incidence of prostate cancer is stabilizing or even decreasing, along with a reduction in cancer-related mortality as a result of improvements in its management [2]. However, it is estimated that more than 10 million men may be living with prostate cancer worldwide, of whom at least 700,000 may have metastatic disease [3].

In recent years, a large amount of data has emerged regarding metastatic hormone-sensitive prostate cancer (mHSPC). According to the so-called CHAARTED (Chemohormonal Therapy Versus Androgen Ablation Randomized Trial for Extensive Disease in Prostate Cancer) criteria, mHSPC is usually categorized as high- or low-volume disease [4]. Low-volume metastatic disease means anything other than that which fits the criteria for high-volume mHSPC, e.g., four or more bone lesions with one or more lesion in any body structure beyond the spine or pelvis, or visceral disease (non-nodal) assessed by bone scan and computed tomography (CT). It is not clear whether oligometastatic disease [5] can be attributed to low- or high-volume disease; it is not clearly defined in current guidelines [6]. Hypothetically, a patient with a single lung metastasis in the absence of bone or nodal involvement could be classified as oligometastatic (one lesion) but high volume (visceral disease).

Patients with high-volume metastatic disease at diagnosis usually have poorer survival outcomes [7,8]. For most patients with mHSPC, a combination of systemic therapies are considered as the standard of care, including androgen deprivation therapy (ADT) as the backbone treatment, adding androgen receptor-targeted agents (ARTAs) [9] and/or docetaxel [10], according to the disease and patient characteristics [11,12]. A long-term survival analysis of the CHAARTED study reported that chemohormonal therapy offered no survival benefit among patients with low-volume disease [13], while no impact of volume on the benefit from the addition of docetaxel was expected from STAMPEDE [10]. However, although ARTAs are effective for the overall mHSPC population; when it comes to low-volume disease, the overall survival benefit with ARTAs versus the former standard ADT is even higher, both for apalutamide (hazard ratio [HR], 0.52; 95% confidence interval [CI], 0.35–0.79) [14] and enzalutamide (HR, 0.66; 95% CI, 0.43–1.03) [15]. The management of the primary tumor improved overall survival in the low-volume group only (HR, 0.68, 95% CI, 0.52–0.90; *p* = 0.007) [16].

ARTAs and radiotherapy are both recognized as a standard of care in mHSPC, with disease volume, overall performance status, and comorbidities as the most important factors to be considered when tailoring treatment [17]. However, there is limited evidence, and thus consensus, on the optimal treatment of the low-volume forms of mHSPC, either for de novo mHSPC and mostly for metachronous disease, as these patients were either absent [18] or only represented as a minority in the trials [15,16]. With the aim of providing clinicians with guidance on best practice, a group of Italian medical and radiation oncologists, experts on prostate cancer, used a round robin variant of the focus group method to generate a series of consensus statements on the management of low-volume mHSPC, mainly applicable for those working in Italy. This relies on an iterative process of consecutive contributions by each participant.

## 2. Materials and Methods

Compared with traditional focus groups, the round robin variant allows each group member to share equally in the group process, thereby ensuring that no one person dominates the discussion [19]. The process was carried out in two virtual sessions moderated by a senior clinical epidemiologist (G.L.P.), expert in gaining consensus among stakeholders (facilitator). In the first session, participants were asked to individually write down various topics believed to be most relevant for daily practice and thus selected for further group discussion. Participants were asked to consider only therapies that are reimbursed by the national health care system and procedures with relevant literature data. Next, each member of the board in turn independently presented his/her views without discussion. The topics were then ranked by the participants and shortlisted. Based on this discussion, the coordinators met virtually on a second date to collate the discussion and consensus reached by the group.

## 3. Results and Discussion

Twenty-five potential areas for discussion were presented by the participants as possible topics of concern in the management of low-volume mHSPC (Table 1). These were ranked to choose three areas for detailed discussion. The three major areas were definition of oligometastatic disease within low-volume disease, treatment of de novo metastatic disease, and treatment of low-volume metachronous disease. Stage IV N1M0 prostate cancer was not included in this consensus discussion.

### 3.1. Definition of Oligometastatic Disease within Low-Volume Disease

The discussion focused on which criteria could be used to define patients with oligometastatic disease. In general, it was thought that oligometastatic disease has characteristics that guide the choice of whether local therapy can be used, and thus it is also necessary to define it through the location and number of lesions [20]. The experts rejected the statement that oligometastatic and low-volume disease definitions completely overlap. Two consensus documents (Advanced Prostate Cancer Consensus Conference and the Italian Association of Radiotherapy and Clinical Oncology and Radiotherapy) both define oligometastatic disease based on bone and lymph node involvement (i.e., ≤3 synchronous metastases involving bone and/or lymph nodes), but not visceral lesions [21,22]. There are questions on whether the definitions of oligometastatic and low-volume disease according to the CHAARTED study [23] might overlap in certain circumstances (i.e., the absence of visceral metastases and three lesions in the spine and/or pelvis or less) and whether patients are candidates for the same treatments (systemic and/or locoregional).

From the discussion, three scenarios were hypothesized: (1) three non-visceral lesions (i.e., bone or lymph node metastases [23]) or less: in this case, most patients could be defined as both oligometastatic and low volume; (2) the presence of four bone or lymph node metastases: in this case, most patients could be considered oligometastatic, but the presence of four bone lesions with one outside the spine or pelvis identifies high-volume disease; (3) five bone or lymph node lesions: some clinical studies exploring radiotherapy included patients in whom up to five lesions were detected; this scenario could indicate high- or low-volume disease and occasionally oligometastatic disease [24]. However, several meta-analyses have shown the rates of survival of patients with less than five lesions and patients with five or more lesions are different [25,26]. Based on these considerations, consensus was reached that disease with up to three non-visceral lesions could be considered both oligometastatic and low volume. Further extension of the definition of oligometastatic to patients in whom four or five lesions are detected could be considered if this potentially leads to better tailoring of treatment.

Regarding staging, the panel recognized that standard imaging (CT and bone scan) is often replaced by new-generation imaging procedures (e.g., choline or prostate-specific membrane antigen [PSMA] positron emission tomography [PET]/CT) [27]. The usefulness of these examinations was also questioned for disease that had been staged with CT and bone scintigraphy [13,14]. As a general rule, the panel stated that molecular/receptor imaging should not be performed in patients who are already staged with conventional imaging. However, choline or PSMA PET/CT may be proposed as primary staging [28]. Limited evidence exists about the survival benefit of improved diagnostic accuracy obtained with choline/PSMA imaging in this setting, and an unequivocal recommendation about this issue is not provided by current guidelines. In clinical practice, many patients are initially staged with PSMA PET or choline; however, the clinician should be aware that pivotal trials (e.g., CHAARTED and TITAN [Targeted Investigational Treatment Analysis of Novel Anti-androgen]) did not use this method for initial staging [27]. Finally, PET is certainly of value in the case of biochemical recurrence where metastasis-directed therapy could be profitable in the treatment strategy [29].

### 3.2. Treatment of De Novo Metastatic Disease

The discussion focused on which patients can undergo radiotherapy on primary tumors and/or metastases and if and when to add additional medical therapy to ADT.

Moderate evidence supports the use of radiotherapy on primary tumors as shown in the STAMPEDE (Systemic Therapy in Advancing or Metastatic Prostate Cancer: Evaluation of Drug Efficacy) trial [16]. In the trial, radiotherapy improved failure-free survival (HR, 0.76; *p* < 0.0001), but not overall survival (HR, 0.92; *p* = 0.266). In addition, failure-free survival was also improved in patients with low-volume disease who received radiotherapy (HR, 0.59; *p* < 0.0001). 

However, it is also reasonable to consider irradiating oligometastatic sites [30], and in this case, PET is needed to clarify which sites should be irradiated. Moreover, a subgroup analysis of the STAMPEDE trial showed an 8% increase in overall survival at 3 years when radiotherapy was performed on the primary tumors in patients with up to three bone metastases [31].

Regarding treatment on distant metastases, according to the experts, the feasibility of this approach depends on the number of lesions (oligometastatic disease), when it is common to perform radiotherapy on the primary tumor, and a metastasis-directed therapeutic strategy [32]. However, it would be necessary to estimate how much of a benefit ablative therapy would have on the metastases in this scenario. If the primary tumor is being treated in low-volume mHSPC, multidisciplinary evaluation should be used to aid in the decision-making process. Thus, with low-volume disease, the primary tumor should definitely be treated and metastases can be locally treated in selected cases.

The discussion then centered around which patients with de novo metastatic and low-volume disease should be administered ARTAs along with ADT. This scenario is changing as a result of the approval of new hormonal agents, which have shown promising results that indicate benefit on both overall and progression-free survival in all subgroups analyzed (e.g., in the TITAN study, the HR for overall survival for de novo metastases and primary progressive tumors was 0.68 and 0.39, respectively; and 0.52 and 0.70 for low- and high-volume disease, respectively) [14,33]. With the availability of the new ARTAs, ADT alone should be considered undertreatment. In patients with de novo metastatic and low-volume disease, the use of ARTAs can dramatically change outcomes. In TITAN, apalutamide plus ADT significantly reduced the risk of death by 48% compared with placebo (HR, 0.52; 95% CI 0.35–0.79) [14]. In ARCHES (NCT02677896), enzalutamide + ADT also extended survival compared with placebo + ADT (HR, 0.66; *p* < 0.0001) [15], and in the ENZAMET trial, enzalutamide + ADT± docetaxel significantly reduced the risk of death in low-volume disease (HR: 0.54, 95% CI 0.39–0.74) [34]. Overall, the tolerability of ARTAs was good, and the patients’ health-related quality of life was maintained [14].

With the availability of ARTAs and considering the data from the STAMPEDE arm H trial [16], the panel was in favor of intensification of therapy. When a limited number of synchronous lesions are detected, the state-of-the-art approach should include ADT + ARTA in combination, with the addition of radiotherapy to the primary tumor (except in selected cases, e.g., very elderly and with important comorbidities for whom a tailored approach is suggested). Metastasis-directed therapy with ablative intent could be beneficial in selected cases.

In summary, adding ARTAs and radiotherapy to ADT should be considered as a valid therapeutic option for any fit patient with de novo low-volume/oligometastatic non-visceral prostate cancer. For this, the therapeutic choice should be individualized through multidisciplinary discussion.

### 3.3. Treatment of Low-Volume Metachronous Disease

The first aspect that was discussed was the criteria that guide the choice of systemic or locoregional therapy, and in the case of ADT, whether ARTA should be added for patients with low-volume metachronous disease.

In general, it was held that it is necessary to evaluate the timing of progression and the patient’s general condition and motivation to eventually postpone ADT. Locoregional treatment can be considered in selected cases, such as those with a high prostate-specific antigen (PSA) doubling time or low PSA levels, slow progression, or the presence of oligometastatic disease by imaging studies. If the disease reappears shortly after previous locoregional treatment, systemic treatment should be initiated without delay. The possible addition of ADT to radiotherapy should be discussed with the patient.

All patients who experience biochemical recurrence with PSA < 1 ng/mL should be restaged with PSMA PET and then receive metastases-directed therapy in the presence of oligometastatic disease according to the definition of the Italian Association of Clinical Oncology and Radiotherapy (i.e., three non-visceral lesions or less) [21]. In this case, ADT could be delayed [35]. If the patient has oligometastatic disease with PSA > 1 ng/mL and is a candidate for radiotherapy on metastatic lesions, a decision is made on the basis of the Gleason score, time to onset, and after discussion with the patient.

In addition to the characteristics of the patient, the choice of therapy must also be guided by the features of the disease, even when the choice is based mostly on clinical experience. Greater biological aggressiveness (based on PSA doubling time, Gleason score, etc.) favors earlier systemic treatment.

In recent years, accessibility to radiotherapy in Italy has improved and targeted ablative treatments are widely available. The panel stated that, in well-selected patients, when biochemical control of the disease is obtained after metastasis-directed therapy, a second course of stereotactic body radiotherapy can be proposed if time to relapse is >1 year, considering data from the STOMP (Surveillance or Metastasis-Directed Therapy for Oligometastatic Prostate Cancer Recurrence) trial [36].

At present, ablation and locoregional treatment of lesions can be performed before systemic therapy. These patients should undergo multidisciplinary evaluation, especially with the advent of ARTAs in clinical practice. The availability of novel hormonal agents has the possibility to change clinical practice, and data on the use of ARTAs has shown benefit in radiographic progression-free survival and overall survival in patients with metachronous mHSPC (i.e., in TITAN, radiographic progression-free survival improved by 59% and overall survival by 61% vs. ADT alone) [14,33].

In the case of metachronous disease, the issue of staging arises, and multidisciplinary evaluation is needed. In pivotal studies, patients were staged with a CT scan and scintigraphy and underwent treatment with ARTA + ADT [14,37]. The benefit of systemic treatment has yet to be confirmed in patients undergoing more sensitive imaging with earlier diagnosis of metastatic disease. In the case of medical therapy given at an early stage, even if administered in the long term, the experts held that safety issues were not a major concern because concomitant treatment does not appear to increase toxicity even in patients > 75 years old, although more careful monitoring may be warranted. However, it was further highlighted that clinicians must also learn to manage long-term toxicities, such as bone health, that were not widely considered in the past.

The final recommendations, with the consensus of the entire expert panel, are summarized in Table 2.

## 4. Conclusions

Based on expert clinical experience, this report had the specific goal of providing recommendations for clinicians in daily practice regarding management of the low-volume forms of mHSPC for which there is scarce evidence-based management. Therefore, a formal consensus method was successfully adopted; a straightforward approach in which all participants were given equal opportunity to express their opinion ensured that interpersonal dynamics were kept under control. Specifically, consensus was obtained on three major areas of controversy: the clinical definitions of mHSPC; therapy for de novo metastatic disease; and therapy for metachronous metastatic disease. It is hoped that clinicians treating patients with mHSPC in daily practice will find this sharing of expert opinion of value.

## Figures and Tables

**Table 1:** Areas considered for discussion after round robin 1.

Adequate Staging	Family History
Low volume = oligometastatic?	Comorbidities
Ablation of all metastatic lesions	Cost/benefit analysis
Role of prostate-specific antigen	Local staging with symptoms and International Prostate Symptom Score
Life expectancy	Availability of drugs
Multidisciplinary management	Concomitant therapies
Treatment of the primary tumor	Histologic variants
Simple vs. complicated lesions	Size of lesions
Role of the Gleason score	Strategies for combining chemotherapy and radiotherapy
De novo diagnosis vs. disease recurrence	Patient preferences
Site of disease	Early treatment
Definition of oligometastatic (no. of lesions)	Tolerability of therapy
Accessibility to radiotherapy	

**Table 2:** Summary of recommendations for definitions of and therapy for mHSPC.

Area	Recommendations
mHSPC definitions	Oligometastatic and low-volume disease refer to different concepts and should not be used interchangeably
De novo metastatic disease	ADT alone can be considered undertreatment Patients should be evaluated for combination systemic treatment (e.g., ADT + ARTA) Local therapy should not be denied in patients with low-volume mHSPC, regardless of the intensification of systemic therapy Metastasis-directed therapy can be proposed in selected cases
Metachronous metastatic disease	Metastasis-directed therapy can be proposed to delay systemic treatment in selected cases (if PSMA PET staging has been performed, high PSA doubling time) Patients should be evaluated for combination systemic treatment (e.g., ADT + ARTA)

mHSPC, hormone-sensitive prostate cancer; ADT, androgen deprivation therapy; ARTA, androgen receptor-targeted agent; PSMA, prostate-specific membrane antigen; PET, positron emission tomography.

## Data Availability

The data presented in this study are available in this article.
